# Management of Turner Syndrome

**DOI:** 10.17925/EE.2015.11.02.100

**Published:** 2015-08-19

**Authors:** Aneta Gawlik

**Affiliations:** Assistant Professor, Pediatric Endocrinology and Diabetes, Department of Pediatrics, Medical University of Silesia, School of Medicine in Katowice, Poland

**Keywords:** Turner syndrome, genetic disorder, healthcare, short stature, puberty induction, cardiac malformation

## Abstract

Turner syndrome is one of the most common genetic disorders, affecting one in 2,000–2,500 live-born girls. In order to provide appropriate healthcare, a multi-disciplinary team of closely cooperating endocrinologists, gynaecologists, geneticists, cardiologists, otolaryngologists, fertility specialists, psychologists, nurse educators and social workers is needed.

The diagnosis of Turner syndrome (TS) is based on a complete or partial absence of the second X chromosome. Structural anomalies of the sex chromosome include isoXq (duplication of the long arm to form an isochromosome), rX (ring formation) and Xp-/Xq- (deletion of the short/long arm). Some individuals can present a mosaic karyotype (45,X/46,XX), also with the Y chromosome (45,X/46,XY). Our knowledge about the genotype–phenotype correlation or the influence of X origin (paternal or maternal) on the phenotype remains poor. What is commonly known is that a patient with X monosomy presents the most severe clinical picture, while in the 45,X/46,XX mosaicism, the TS phenotype is milder. TS can be diagnosed only in phenotypic females presenting some characteristic features. Importantly, not all patients with the 45,X/46,XY karyotype, but only those with the female phenotype, can be recognised as having TS. The most important factors in TS are short stature, cardiovascular disorders (cardiac defects and hypertension), otolaryngological problems (hearing loss), delayed or lack of puberty as a symptom of gonadal failure, renal anomalies, obesity and metabolic disorders, autoimmune diseases, some behavioural difficulties and, at an older age, osteoporosis.^1^

TS patients require multi-disciplinary and long-term medical healthcare. At various ages, different medical problems are the focus of treatment. Short stature is diagnosed in approximately 95 % of TS patients. Without medical intervention, the final height is 20 cm below normal range.^2^ Haploinsufficiency of short stature homebox *(SHOX)* gene is responsible for short stature and other TS-associated skeletal anomalies.

The efficacy of recombinant growth hormone (GH) therapy was first confirmed by a Canadian study.^3^ Positive predictive markers of good response to GH include tall height at therapy onset, taller parents, better first-year responsiveness to GH, higher GH doses and longer time of therapy; the optimal age for the start of GH treatment has not as yet been established. Bone age more than 14 years and/or growth velocity less than 2 cm/year indicate the end of growth-promoting therapy. In some older patients (~9 years) with very poor prognosis of final height, the addition of a low dose of oxandrolone (non-aromatisable anabolic steroid) should be considered.^4^ The addition of Ox to GH treatment may increase adult height by even more than 4 cm.^5^

**Table 1:** Monitoring Algorithm in Turner Syndrome Patients^1^

Monitoring (Irrespective of Symptoms)	<5 Year	School Age	Older Patients
Cardiological evaluation	x	x	x
Blood pressure annually	x	x	x
Audiology every 1–5 years	x	x	x
Social skills at age 4–5 years	x		
Liver and thyroid screening annually		x	x
Coeliac screen every 2–5 years		x	x
Social and educational progress annually		x	
Dental and orthodontic as needed		x	
Lipids and blood sugar annually			x
Evaluation for growth	x	x	
Age-appropriate evaluation of pubertal development and psychosexual adjustment		x	x

The lack of pubertal growth spurt is one of the causes of short stature in TS patients. Gonadal dysgenesis is diagnosed in most TS patients; however, more than 30 % of girls with TS present some symptoms of puberty (especially patients with the 45,X/46,XX karyotype). If growth-promoting therapy is initiated at a younger age, puberty induction can be started earlier in order to mimic physiology. The mean age for oestrogen therapy is 12 to 14 but low-dose treatment can be started even in younger girls.^1^ Puberty should be initiated by using low-dose transdermal oestrogens, although the optimal formulation, dosage and route of administration are still under dispute. Hormone replacement therapy (HRT) should be continued until the time corresponding to normal menopause. Unfortunately, recent studies have shown that a considerable percentage of women with TS discontinue HRT in adulthood.^6,7^

Spontaneous pregnancy is possible in only 2 % of TS women. The preparation of a TS patient for pregnancy, either spontaneous or by *in vitro* fertilisation (IVF), should include a risk assessment of potential pregnancy-related complications in TS. Beside the regular thyroid function and glucose tolerance tests, cardiovascular and renal system evaluation is also recommended. The risk of aortic dissection or rupture during pregnancy in TS women with cardiac defect is high^8^ and can occur without cardiac malformation or hypertension.^9^

The quality of life in TS patients is affected by a higher incidence of autoimmune diseases and hearing problems. Repeated screening for autoimmune thyroid diseases (Hashimoto thyroiditis and Graves’ disease), coeliac disease and diabetes mellitus is recommended. Since more than half of adults with TS experience hearing problem, regular otological examination is crucial.^10^

TS patients require lifelong high-quality and developmentally adequate healthcare (see *Table 1*). Early diagnosis, the cooperation of a multidisciplinary team familiar with TS-related health problems as well as the patient’s understanding of the main factors associated with this genetic disorder are essential for successful patient management.
